# Mediterranean Diet and Nutrition Literacy: Translating Evidence into Food Choices, Health, and Sustainability

**DOI:** 10.3390/nu18121901

**Published:** 2026-06-12

**Authors:** Paula Silva

**Affiliations:** 1 Department of Microscopy, School of Medicine and Biomedical Sciences (ICBAS), University of Porto (U. Porto), Rua Jorge Viterbo Ferreira 228, 4050-313 Porto, Portugal; psilva@icbas.up.pt; 2 iNOVA Media Lab, ICNOVA-NOVA Institute of Communication, NOVA School of Social Sciences and Humanities, Universidade NOVA de Lisboa, 1069-061 Lisbon, Portugal

The Mediterranean Diet is one of the most extensively studied dietary patterns in nutritional science and public health. Its relevance extends beyond disease prevention; it is also a cultural, culinary, social, and environmental model rooted in local food systems, seasonal foods, traditional practices, and commensality (eating together). However, despite its recognized value, adherence to the Mediterranean Diet has reduced in several Mediterranean settings, particularly among younger generations and socioeconomically vulnerable populations [1,2,3]. This paradox defines one of the major contemporary challenges in nutrition: the question is no longer whether the Mediterranean Diet is beneficial, but how scientific evidence can be translated into sustained, meaningful, and equitable food practices.

This Special Issue, “Mediterranean Diet and Nutrition Literacy,” brings together five contributions that address this challenge from complementary perspectives [1,2,4,5,6]. Collectively, they position the Mediterranean Diet not merely as a set of foods or nutrients but as a knowledge-to-practice framework connecting biological mechanisms, dietary behaviors, literacy, food environments, sustainability, cultural context, and public health intervention. This integrated view is consistent with contemporary approaches to food literacy, which emphasize that healthy eating depends not only on knowledge but also on competencies related to planning, selecting, acquiring, preparing, cooking, and critically evaluating food-related information [7].

A central message emerging from this Special Issue is that nutrition literacy should be understood as an applied capacity. This includes the ability to understand, interpret, evaluate, and use food-related knowledge in daily life [2,6,7]. This was particularly evident in a study conducted among women aged 45–70 years living in socioeconomically disadvantaged neighborhoods in Florence. In this population, nutrition literacy was positively associated with adherence to the Mediterranean Diet, and the “Food Label and Numeracy” dimension emerged as an independent predictor of moderate or high adherence [2]. This finding shifts the focus from abstract nutritional knowledge to concrete competencies, such as reading labels, interpreting quantities, understanding portions, and navigating food information in routine decisions.

This interpretation is reinforced by a study investigating participants’ knowledge of the health benefits of fruits, vegetables, and antioxidants, adherence to the Mediterranean Diet, and propensity toward sustainable food purchasing [6]. Although the participants showed a generally adequate level of knowledge, this did not automatically translate into strong sustainability-oriented purchasing behaviors. Simultaneously, knowledge of the benefits of fruits and vegetables, and of antioxidants, was positively associated with sustainable purchasing [6]. These findings illustrate the complexity of the knowledge-to-action pathway. Knowledge matters, but it is insufficient when detached from practical skills, motivation, food access, affordability, cultural relevance, and the capacity to act within specific food environments [2,6,7,8].

This gap between knowledge and practice is especially relevant during adolescence, when food preferences, autonomy, identity, and long-term habits are shaped [1,3,8]. Evidence from early adolescents shows that higher nutrition literacy is associated with greater adherence to the Mediterranean Diet and healthier lifestyle behaviors, including a lower screen time [3]. Similarly, recent research among adolescents has found positive associations between nutrition literacy, Mediterranean Diet compliance, sustainable environmental attitudes, and ecological footprint-related indicators [9]. These findings support the idea that food and nutrition literacy during adolescence may operate at the intersection of health, sustainability, and agency.

The FOODWISELab: The Mediterranean Diet Experience article addresses this challenge by proposing a culturally grounded, school-based model designed for the Portuguese educational context [1]. Its relevance lies not only in its focus on adolescents but also in its pedagogical architecture. Organized around planning, selecting, preparing, and eating, the intervention translates the Mediterranean Diet into a set of lived practices [1]. School gardens, cooking activities, digital tools, family engagement, and community links transformed dietary guidance into experiential learning. This approach is aligned with broader evidence showing that children’s and adolescents’ eating behaviors are shaped by multiple interacting factors, including individual preferences, food literacy competencies, family practices, school environments, external food environments, and the food supply chain [8].

The importance of school-based approaches is also supported by systematic evidence showing that schools are critical interfaces for nutrition promotion among children and adolescents [10]. However, this literature also reveals that school nutrition interventions are heterogeneous and that stronger evidence is needed regarding their implementation, equity, sustainability, and long-term outcomes [10]. In this context, FOODWISELab exemplifies a broader principle that runs across this Special Issue: Mediterranean Diet promotion must move from prescription to participation.

The biological foundations of this dietary model have been addressed in a review on extra virgin olive oil’s phenolic compounds and mitochondrial function [4]. Extra virgin olive oil is not only a symbolic marker of the Mediterranean Diet but also a biologically active food matrix. This review highlights the role of phenolic compounds, including hydroxytyrosol, oleuropein, and oleocanthal, in oxidative stress, inflammation, mitochondrial biogenesis, electron transport chain efficiency, and mitochondrial DNA protection [4]. These mechanisms help explain how traditional foods may contribute to chronic disease prevention and healthy aging. Nevertheless, they also create communication challenges. If the public health value of the Mediterranean Diet is to be understood and adopted, complex biological knowledge must be translated into clear, accurate, and usable messages without reducing the diet to isolated compounds or simplistic health claims [2,4,6].

The article on vegetable intake and metabolic dysfunction-associated steatotic liver disease offers another bridge between evidence and practice [5]. By examining the total vegetable intake and vegetable subgroups in a South Italian cohort, the study provides more precise information on the quantity and type of vegetables associated with a lower MASLD risk [5]. This finding is particularly relevant for nutrition literacy because it shows the need to move beyond generic messages such as “eat more vegetables.” Public health communication should help people understand what kinds of vegetables, in what amounts, prepared in what ways, and within which dietary patterns, are the most relevant for health [2,5,6].

The health relevance of promoting the Mediterranean Diet from early life is further supported by a recent systematic review and meta-analysis of randomized clinical trials showing that Mediterranean Diet-based interventions may improve cardiometabolic biomarkers in children and adolescents, including systolic blood pressure and lipid profile parameters [11]. This evidence reinforces the need to integrate dietary education, literacy, and prevention strategies earlier in the life course.

Across these contributions, the Mediterranean Diet has emerged as a multidimensional model linking food quality, bioactive compounds, dietary behavior, cultural identity, sustainability, and disease prevention [1,2,4,5,6]. The articles also show that promotion cannot rely on information dissemination alone. It requires interventions adapted to different life stages and social contexts: adolescents in schools, women at increased cardiometabolic risk, adults making purchasing decisions, and populations exposed to metabolic disease risks [1,2,5,6]. It also requires attention to the digital environment. Recent evidence suggests that digital healthy eating literacy is associated with environmentally responsible food choices and adherence to the Mediterranean Diet, indicating that future nutrition literacy strategies must also address the quality, accessibility, and credibility of online nutrition information [12].

Therefore, this Special Issue contributes to an important reframing of the Mediterranean Diet research. The field should move beyond asking whether the Mediterranean Diet is healthy toward a more complex research agenda: how people understand it, how they access it, how they adapt it to contemporary food environments, and how they can be supported in practicing it across the life course. Future studies should prioritize longitudinal and intervention designs capable of clarifying the causal pathways between nutrition literacy, dietary adherence, sustainability-related behaviors, and health outcomes [2,3,6,9]. Particular attention should be given to the most actionable dimensions of nutrition literacy, including food label interpretation, numeracy, meal planning, food preparation, digital information appraisal, and critical evaluation of nutrition claims [2,7,12].

Further research is needed to evaluate the scalability and effectiveness of school-based, community-based, and digitally supported interventions [1,10,12]. Models such as FOODWISELab are promising because they combine evidence-based content with experiential learning and cultural relevance [1]. However, their long-term impacts on food literacy, family practices, sustainability awareness, dietary behavior, and health indicators should be rigorously assessed. Simultaneously, mechanistic studies on key Mediterranean foods, such as extra virgin olive oil, should be better integrated with behavioral and communication research [4]. Understanding biological pathways is essential, but the public health impact depends on whether this knowledge can be translated into recommendations that people can understand, trust, and apply.

In conclusion, the five papers included in this Special Issue collectively show that Mediterranean Diet and nutrition literacy research is entering a more integrated phase [1,2,4,5,6]. The Mediterranean Diet should be promoted not as a nostalgic return to the past or as a narrow nutritional prescription but as a living, adaptable, evidence-based framework for health, sustainability, and cultural continuity. Nutrition literacy bridges scientific evidence and daily food choices. Strengthening this bridge is one of the most important tasks in future nutrition research, education, and public health practice.
